# Potential persistence mechanisms of the major *Anopheles gambiae* species complex malaria vectors in sub-Saharan Africa: a narrative review

**DOI:** 10.1186/s12936-023-04775-0

**Published:** 2023-11-07

**Authors:** Rita Mwima, Tin-Yu J. Hui, Ann Nanteza, Austin Burt, Jonathan K. Kayondo

**Affiliations:** 1 Department of Entomology, Uganda Virus Research Institute (UVRI), Entebbe, Uganda; 2 Department of Biotechnical and Diagnostic Sciences, College of Veterinary Medicine, Animal Resources and Biosecurity (COVAB), Makerere University, Kampala, Uganda; 3 Silwood Park Campus, Department of Life Sciences, Imperial College London, Ascot, UK

**Keywords:** *Anopheles*, Persistence mechanisms, Dry season survival, Malaria

## Abstract

The source of malaria vector populations that re-establish at the beginning of the rainy season is still unclear yet knowledge of mosquito behaviour is required to effectively institute control measures. Alternative hypotheses like aestivation, local refugia, migration between neighbouring sites, and long-distance migration (LDM) are stipulated to support mosquito persistence. This work assessed the malaria vector persistence dynamics and examined various studies done on vector survival  via these hypotheses; aestivation, local refugia, local or long-distance migration across sub-Saharan Africa, explored a range of methods used, ecological parameters and highlighted the knowledge trends and gaps. The results about a particular persistence mechanism that supports the re-establishment of *Anopheles gambiae*, *Anopheles coluzzii* or *Anopheles arabiensis* in sub-Saharan Africa were not conclusive given that each method used had its limitations. For example, the Mark-Release-Recapture (MRR) method whose challenge is a low recapture rate that affects its accuracy, and the use of time series analysis through field collections whose challenge is the uncertainty about whether not finding mosquitoes during the dry season is a weakness of the conventional sampling methods used or because of hidden shelters. This, therefore, calls for further investigations emphasizing the use of ecological experiments under controlled conditions in the laboratory or semi-field, and genetic approaches, as they are known to complement each other. This review, therefore, unveils and assesses the uncertainties that influence the different malaria vector persistence mechanisms and provides recommendations for future studies.

**Supplementary Information:**

The online version contains supplementary material available at 10.1186/s12936-023-04775-0.

## Background

Malaria vector populations exhibit strong seasonal fluctuations in abundance and are present in large numbers during the rainy season, but drop to extremely low levels when the larval habitats dry up [1–3]. This has been observed within members of the *Anopheles gambiae* species complex (or *Anopheles gambiae *sensu lato) (Diptera: Culicidae) and beyond, and across diverse ecological or geographical set-ups, including the West-African Sahel and East Africa Savanna. Prevailing hypotheses suggest that the possible ways that could explain the seasonal malaria mosquito population dynamics are: (1) local mosquito populations experience dry season bottlenecks and are sustained by a few hidden survivors (aestivation) [4]; (2) local populations become extinct and few migrants from neighbouring areas, where permanent breeding occurs, recolonize the area at the beginning of the rainy season (local migration) [5, 6]; (3) the local population gets extinct during the dry season and is recolonized by long-distance migrants from stable areas (long-distance migration, LDM) [7]; and (4) large populations survive locally but are hidden with respect to sampling methods (also known as hidden or local refugia) [8, 9].

Despite the findings at hand from different studies, the source of malaria mosquito populations that re-establish at the start of a rainy season remains a mystery mostly because getting direct evidence of adults in their hidden shelters or even recapturing marked mosquitoes around the release sites is difficult [4, 10]. Genetic studies have been conducted to test whether populations undergo annual dry season bottlenecks [11, 12], but have not yielded conclusive results. This could be because of the type of loci that are targeted, using an insufficient number of loci that negatively impacts the statistical power, unavailability of mosquito samples with longer alternating time series, using limited sample collection methods (which are not representative of both endophilic and exophilic fractions of a particular population to account for behavioural heterogeneity and aid in estimating total effective population size (Ne)), and no knowledge of how selection affects allele frequency changes and consequently Ne estimates [2, 11–13].

Here, it is essential to distinguish between the persistence mechanisms used by malaria vector species in either the Equatorial or Sahelian regions. It is important to note that in the Equatorial region, there could always be surface water available nearly all year round or the dry season could be short relative to their life cycle (e.g. less than 2 months). Therefore, mosquito persistence mechanisms might not be required, or could be by local migration or local refugia. In the Sahelian region on the other hand, there  is never surface water in vast areas spanning the long dry season that usually lasts between 3 and 8 months.

**Fig. 1:**
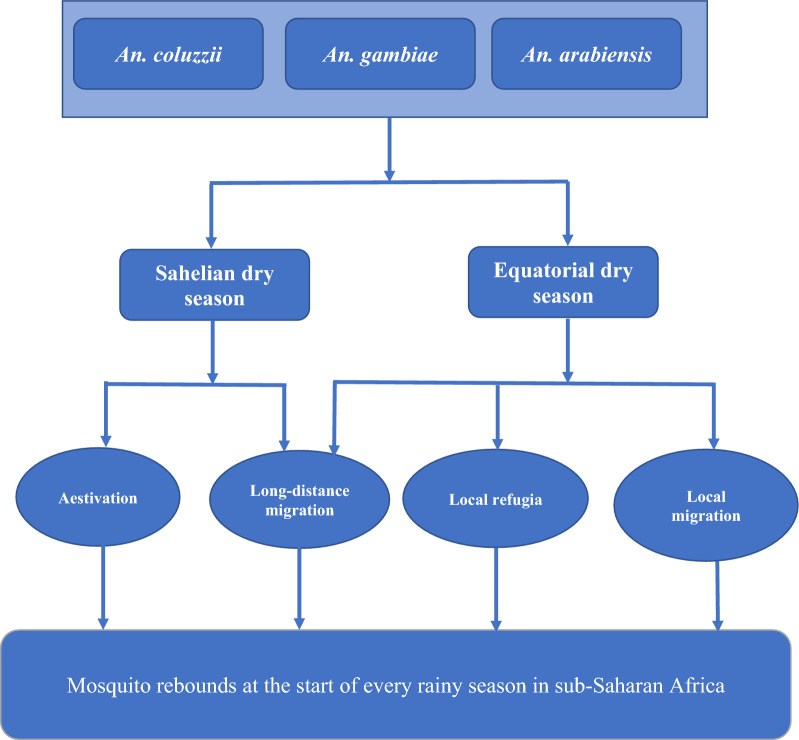
Schematic diagram showing the different persistence mechanisms responsible for the early rainy season malaria mosquito rebounds across sub-Saharan Africa. The four hypotheses could be responsible for population rebounds of the *An. gambiae *species complex at the start of each rainy season

The exact persistence mechanisms used by malaria vector species in sub-Saharan Africa (Fig. 1) is a conundrum, given that the four hypotheses explain the rapid mosquito rebounds at the beginning of each wet season [4, 12, 14]. Various studies concerned with which populations contribute to the early rainy season malaria mosquito rebounds have been carried out, and in this review, their strengths and weaknesses will be accessed based on the study design, the methods used and whether the conclusions support the results, and thereafter highlight the gaps that remain therein (Table 1). This review, therefore, focuses on the uncertainties of the persistence mechanisms utilized by malaria vectors across sub-Saharan Africa.

**Table 1:** Summary of studies on persistence mechanisms of malaria mosquitoes in sub-Saharan Africa

Method, persistence mechanism being tested & reference	Strengths	Weaknesses	Key assumptions	Comments
Mark Release Recapture (MRR) to determine whether malaria mosquitoes survive the dry season by aestivation [4, 10, 63, 80]	Informs population size, survival rate and movement May be the only method that can provide unequivocal proof for aestivation and migration	Affected by the dry season with few or no mosquitoes Without sequencing of recaptured mosquitoes, results are not confirmatory (aestivation/local refugia) Low recapture rate, thus affecting the accuracy of the method Does not reveal where mosquito shelters are and how they cope with the dry season	The marked mosquitoes become re-integrated into the rest of the population Marking mosquitoes does not adversely affect them Mortality of marked mosquitoes caused specifically by their recapture is ignored Mortality rate is constant throughout	The aestivation process is difficult to reproduce Some females break their aestivation more readily than others
Lab studies to determine whether malaria mosquitoes survive the dry season by aestivation [81]	In this study, the maximum lifespan of *Anopheles* mosquitoes was over 100 days representing maximum longevity compared to standard insectary conditions by 2.2–3.5-fold	Laboratory colonies lose genetic diversity in a few generations Laboratory conditions do not recapitulate all of the possible cues present in the field The lack of unambiguous markers of aestivation in *Anophelines* made it difficult to clearly confirm whether it really happened Demonstrating aestivation in its entirety in the lab is still a challenge [81]	This study used somewhat exaggerated climatic conditions to induce longevity with reduced temperature and photoperiod	Using exaggerated photoperiods beyond what happens in Mali is likely to have pushed *An. gambiae* to have similar longevity to that of *An. coluzzii*, something studies carried out to date have not reported
Field collections to confirm whether mosquitoes survive by aestivation or as local refugia [8, 14, 28, 31, 52]	No specific functional approach [82] Areas with determinants of high mosquito density are established to show sources of dry-season populations	Vector density too low during dry season Distinguishing between absences that are a result of poor sampling and those which are legitimate is a challenge	Changes occur in mosquito physiology and behaviour in dry season Ovaries undergo one gonotrophic cycle in dry season and develop slowly	In dry season, females occupy hidden habitats Low temperatures and relative humidity induce a state of arrested development
Time series analysis (Field collections) to confirm whether mosquitoes survive by aestivation or migration [7]	More reliable results as mosquitoes are collected over a relatively long period of time	Not clear whether not collecting mosquitoes during dry season is a weakness of the sample collection method or because of hidden shelters	When the mosquito recapture rate was less than 3%, the effect of removing them from the subsequent density instead of releasing them was negligible	Climate is one of the Selective pressures responsible for the ecological divergence between *An. coluzzi*i and *An. gambiae* species The *An. arabiensis* collected during the dry season Could be representative of backcrossed hybrids between *An. coluzzii* and *An. arabiensis*
Aerial sampling of mosquitoes at 40-290 m above ground level to confirm whether mosquitoes undergo long distance migration [29]	Results disprove previous studies that malaria mosquito dispersal doesn’t exceed 5 km [63, 64]	There is need to separate the role of Odyssean malaria from windborne migrants Protocol optimisation is time consuming, takes close to 12 months	Mosquitoes ascend by their own flight but are also passively carried by wind atltitude Mosquitoes fly in a layer between 50 and 250 m above ground level (and probably higher) Mosquito flights started at or after 18:00 and ended by 06:00 the following morning LDM-based migrants remain viable /reproductively fit	The likelihood of capturing *Anopheles* species increased with altitude Malaria mosquitoes migrated over tens to hundreds of kilometres in a single night Females outnumbered the males collected (4:1)
Semi-field study (SFS) to test whether malaria mosquitoes survive the dry season by aestivation or migration [83]	SFS bridge the conceptual and methodological gaps between laboratory and field experiments Lifecycle completion is feasible inside the SFS	Laboratory colonies do not represent the wild type as they lose genetic diversity in a few generations A few larvae are sampled to avoid population depletion The hidden mosquito shelters used give a biased representation of the natural environment	Aestivation and migration are the main mechanisms that explain variation in population dynamics	Study results showed that *An. coluzzii* and *An. arabiensis* aestivate while *An gambiae* could adopt a different dry season survival strategy such as LDM Host feeding preferences could be involved in causing species variation of the SFS
The indirect approach: Using genetic data [3, 6, 12, 55]	The method used is sensitive to bottlenecks of population size (robust) Ne depends on both population density and patterns of movement Additional inference based on inter-annual and inter-seasonal changes in private alleles and other measures of pop genetic constitution may be key to identify continuation of breeding vs. migration	Reliable estimates of Ne are difficult to obtain for natural populations Violation of the assumptions considered could result in larger Ne values More information is required to assess the effect of constraints on Ne estimates Ne is not meaningful if we don’t know the geographical area it represents and the population structure model these species follow	Random mating between individuals, discrete generations, a sex ratio of one, negligible selection, migration and mutation	Large populations are maintained throughout the dry season Large populations could be maintained by individuals hidden with respect to sampling Large populations could be maintained by extensive movement of adults

### *Anopheline* mosquitoes in sub-Saharan Africa

In sub-Saharan Africa, the main groups of malaria vectors are *An. gambiae, An. coluzzii*, *An. arabiensis*, and *Anopheles funestus* [15, 16], which are genetically distinct [17]. *Anopheles gambiae* and *An. coluzzii* were once considered as one species until recently. They remain as part of the *An. gambiae* species complex alongside *An. arabiensis*, hence are morphologically inseparable. It is worth mentioning *An. funestus*, which belongs to its own group of species [18–20]. The four are amongst the most efficient, broadly distributed, and dominant malaria vectors in sub-Saharan Africa. These species inhabit diverse environments that include areas where the water that is required for larval development is absent for more than 4 months [4]. Their bionomics vary according to species and in several aspects such as biting rates, duration of their gonotrophic cycles, fecundity, survival, and development of immature and adult stages. *Anopheles arabiensis* lives in dry savannah environments but occupies similar larval habitats to *An. gambiae* [21], thus, occurs in sympatry [19] with their relative abundance dependent on local ecological conditions [22]. It is said that “*An. gambiae* is predominantly anthropophagic and endophilic, and together with its longevity, has a higher vectorial capacity than other species of the *An. gambiae* species complex” [22].

The *An. gambiae* species complex is the major malaria vector characterized by endophagy (preferences for obtaining blood meals indoors), anthropophily (blood meals from humans), and endophily (indoor resting following blood meals) [3]. Its distribution spans most of sub-Saharan Africa and can survive under a wide range of ecological, geographical and seasonal conditions [22]. *Anopheles coluzzii* has high ecological plasticity; thus, it can exploit different habitats [23, 24] and has an opportunistic host-seeking behaviour [25].

However, *An. arabiensis* is known for its ecophenotypic plasticity and is predominantly exophagic (feeds outdoors) and exophilic (rests outdoors) [22]. Because of its ability to develop in residual pools of water in dry riverbeds, it can survive arid conditions and in turn rapidly become abundant at the onset of rains [22].

### The biology of malaria mosquito persistence

The *Anopheline* mosquito populations withstand dry conditions which could last three to 8 months [26], equivalent to several generations of their life time [27]. The hypotheses that explain malaria mosquito persistence mechanisms are aestivation [4], persistence at local refugia [8], local migration [28] and LDM [29].

Aestivation is a repeated state of summer dormancy that constitutes suppressed reproduction and growth in order to ensure extended mosquito survival during the harsh conditions of the dry season [4]. Local refugia populations are those that have the ability to survive under adverse conditions, but remain hidden with respect to conventional sampling methods and can only be found by actively searching for them [8]. Local migration involves mosquito movement from adjacent areas, while LDM is the movement of mosquitoes to favourable areas from further fields, potentially hundreds of kilometres away and is predominantly wind-aided [29].

The mechanisms by which *An. gambiae* species complex persist throughout the dry season vary from the Equatorial to Sahelian region across sub-Saharan Africa [30]. Unlike the Sahelian region, the Equatorial region experiences a milder dry season during which some larval sites remain available within a 5–10 km radius [8, 31]. These few but constantly available larval sites during the dry season are known to act as a strong selection force against aestivation as the persistence mechanism used [32]. Instead, refugia populations are said to occupy distinct hidden habitats during the dry season, which sites could be difficult to detect using conventional sampling methods [8].

Distinguishing between whether the lack of direct evidence for aestivating females during the dry season could be because of the weakness of conventional sampling methods or total absence is very difficult [8]. However, the difference between aestivating mosquitoes and those maintained as refugia is that aestivating females become gonotrophically discordant, and could either fail to develop eggs after taking a blood meal [33], or because they lack suitable oviposition sites, do not lay eggs, but instead, dissolve them and use that as their source of energy [34], while refugia populations continue to breed. It is thought that they can still be found by actively searching for them [8].

It is believed that aestivation is predominantly activated by the absence of water at all the stages of malaria mosquito growth [35]. The eggs of *Anopheles* mosquitoes cannot survive more than 15 days on dry soil [36], therefore, with several months without rain or surface water, it provides the most possible route for survival [35]. During the dry season, malaria vectors generally become susceptible to water loss caused by increased evaporation rates through their spiracles and cuticles [37]. This water loss is linked to reduced survival and oviposition [38], reduced nutritional reserves and egg production [39] and changes in macrogeographic and microgeographic distributions [40].

Dehydration stress has over a period of time resulted in genetic alterations and behavioural adaptations that interact with mosquito physiology, survival and distribution [40]. This could imply that these species experience fitness trade-offs deduced from the fact that, the 2La inversion is associated with higher desiccation resistance and is high in frequency (higher fitness traits) among *An. gambiae* and *An. coluzzii* populations found in arid areas; however, this is rare or even absent in areas where water is readily available [40]. The 2La chromosomal inversions are reported to drive the cuticle thickness and cuticular hydrocarbon (CHC) composition that are responsible for the desiccation-resistant phenotype [40]. Within the *An. gambiae* species complex, dry season metabolic characteristics are evidently similar but show that suppression in metabolic and reproductive processes support the adaptive potential to survive by changing their cuticular, metabolic and behavioural traits [41].

In a genome-wide laboratory-based survey of *An. gambiae* species complex populations, 33 *An. gambiae* desiccation-responsive genes that exhibited reduced transcript accumulation when mosquitoes were exposed to the desiccation treatment and 50 desiccation-responsive genes with known metabolism-related functions altered in response to dehydration were identified [42]. The results from this survey also showed that the number of genes expressed is dependent on the duration of desiccation stress [42]. *Anopheles gambiae* and *An. coluzzii* in particular are known to have the 2La and 2Rb chromosomal inversions [40], which could be associated with aestivation, body size [43] and dry season survival mechanisms [44].

In addition to 2La and 2Rb chromosomal inversions, the *An. gambiae* species complex has other inversions and combinations (2Rc, 2Rd and 2Ru) that are said to be non-randomly correlated with adaptations to arid conditions [45]. These inversions are controlled by the environment and could contribute to local adaptation, habitat range, and desiccation tolerance [40, 46, 47], and may also influence some of the variations in competence for *Plasmodium* [47]. Inversion polymorphisms among local populations could temporally change depending on the seasonal dynamics [48], which explains how various molecular forms of *An. gambiae* species complex develop acclimatization to dry season and increased survival [41].

However, apart from the genetics, because of the high rates of evaporation through their respiratory spiracles and cuticle, mosquitoes are predisposed to water loss which they could deal with by employing several behavioural adaptations, and altering their body size, metabolism and cuticular hydrocarbon composition [37, 39, 49, 50]. Phenotypic differences such as adult body size, reproductive output and longevity could indicate that malaria mosquito molecular forms are adapted to specific niches [24].

The adult *Anopheles* mosquito has a lifespan of less than a month however, some studies indicate that they could survive for over 3 months during the dry season [4, 7, 35, 51]. Results from the studies that have been carried out in the *An. gambiae* species complex on how they survive for more than 4 months of harsh dry season conditions have showed that compared to the wet season; there was a dramatic extension of lifespan [4, 52], they were reproductively suppressed in a state of gonotrophic dissociation [33]; had a 70% reduction in reproduction (between the wet and dry season, the oviposition rate dropped from 70 to 20%, the mean number of eggs per female reduced from 173 to 101 and gonotrophic dissociation increased from 5 to 45%) [51], an 80% reduction in flight activity and the metabolic rate was highest during the dry season [53].

A key feature of aestivation is that it involves a pre-programmed suite of physiological changes that occur in response to one or more external cues such as changes in photoperiods and high temperatures that predict future environmental changes and trigger certain changes in the mosquito to enable it to survive [54]. For mosquitoes in the Sahelian region, the primary forces known to drive aestivation are (1) the absence of surface waters for larval site development (2) temperature fluctuations (3) changes in relative humidity which could confine flight to certain parts of the night [32]. This means that mosquito behavioural changes in selecting suitable microhabitats, suitable times of activity and rest may actually contribute to physiological changes and not necessarily rely on them [32]. Other behavioural changes that are said to occur during the dry season include modification of their feeding habits by switching from human blood to other sources, such as flower nectar and woody-plant juices [55], which are low in protein and could in part be the reason for gonotrophic dissociation that is observed in aestivating adults [32, 51, 53].

In addition to that, when anticipating the coming dry season, *An. coluzzii* have been observed to nearly disappear from villages approximately  one month before the larval sites dry up [4, 14, 51, 53].The work by Huestis and Lehmann [32] hypothesises that behavioural changes in selecting suitable microhabitats in shelters and suitable periods of activity and rest, play a large role in complementing physiological changes, rather than relying on them completely, as is the case for winter diapause.

This can also be supported by the results from the Magombedze et al. [27] study in which two selection bottlenecks that drive phenotypic plasticity occurred: at the beginning of a dry season and selected for mosquitoes able to survive the long dry season, and at the start of the new wet season. These results were comparable to other studies that suggest that malaria mosquitoes in the Sahel region do not use inherited traits (mosquito adaptation) to survive ever-changing environmental conditions, but instead employ a phenotypic switch [56–58].

When reproductive depression was assessed in *An. coluzzii* populations from the Sahel region, the results showed marked seasonality in the reproductive physiology, a drop in response to oviposition, and increased gonotrophic dissociation, which are signs that support survival throughout the dry season by aestivation [51]. Depressed reproduction is, therefore, the most fundamental feature of diapause in adult insects [51], which generally means that for aestivating mosquitoes, during the long dry spell, resources are diverted from reproduction to survival [51].

The key changes noted to happen during the dry season are (1) reduced reproduction [51], (2) reduced flight activity [53], (3) increased tolerance to desiccation attributed to changes in cuticular hydrocarbons [26], and (4) metabolic and protein changes [59].

*The major Anopheles gambiae* species complex malaria vectors are said to undergo these changes only in response to certain external stimuli or cues such as changes in photoperiod, temperature and moisture availability among others that predict the beginning of an environmental change [32]. The cues that have predictors are better suited to initiate aestivation while those without may instead reinforce or maintain it [32]. For example, changes in moisture content (disappearance of larval sites) are a result rather than predictor of a dry season while changes in photoperiod are a predictor that a change in day lengths has occurred and, therefore, initiate aestivation [32]. Case in point was when the responses of *An. coluzzii* and *An. arabiensis* to changes in photoperiod and temperature were compared under dry season conditions, results showed that longevity, body size and total lipids of *An. coluzzii* increased, while those for *An. arabiensis* decreased, a signal that *An. coluzzii* entered the diapause initiation phase [60].

So, given that *An. gambiae* species complex are highly sensitive to temporary oviposition-site deprivation, even dry spells that last just a few days during the wet season can reduce reproductive success [61]. This means that their physiology modifies the effect of oviposition-site deprivation on their reproductive output [61], and because oviposition is largely controlled by water availability with contribution from humidity and rainfall [62], not finding suitable larval sites may be an indication used by mosquitoes to switch from their reproductive state to reproductive depression during the dry season [8, 28, 51, 60].

The wind-aided LDM is the other mechanism by which *An. gambiae* species complex persist through the dry season. So far, studies show that LDM takes place in both Equatorial and Sahelian regions as a means of survival for members of this species [6, 29].

However, from earlier studies carried out in the Sahel, there was scepticism on whether the surge in population was really an indication of migrants from the neighbouring areas or whether they were hidden in the same locality [4]. This was because the neighbouring villages could not serve as a source of migrants, and given that there were low densities of adults throughout the whole area, the Sahelian villages were isolated [4, 14] with studies at that time pointing to the fact that mosquito dispersal over a distance of 2–3 kilometres was unusual [63, 64]. However, an extensive aerial sampling experiment of mosquitoes at 40–290 metres above ground level confirmed the occurrence of windborne migrations among malaria vectors and was estimated to span tens to hundreds of kilometres in a single night [29].

The same study collected 23 *An. coluzzii,* but only 1 *An. gambiae* among the 235 *Anopheline* mosquito migrants, something that contradicted the initial predictions that *An. coluzzii* solely survive the long dry spell by aestivating locally and not through migration in the Sahelian region [4, 7]. *Anopheles coluzzii* could, therefore, survive the long dry spells in the Sahel region by aestivation accompanied by long-distance migration that is said to take place in the late rainy season, otherwise, without migration, the small Sahelian population that survives the dry season through aestivation would become locally extinct [12] because of the unpredicted dry spells that occur during the rainy season [65–67]. This attests to the complexity of species, presenting two strategies that seemed to most as mutually exclusive.

Following wind-borne migration, the ability of each migrant to arrive at a favourable habitat is influenced by changing windspeeds and direction together with the distribution of habitat patches [68]. Migrants could be displaced over hundreds of kilometres in one night, and this may happen for several days [69], depending on the flight capacity and the flight period [68]. The key predictors of long-distance migration include; (1) extinction of the local population during the dry season followed by an abundant rise in population by migrants from areas with favourable climatic conditions that maintain larval sites, (2) the genetic make-up of migrants that arrive at from other areas at the start of the rainy season will be distinct from that of the previous dry season, and (3) when populations are sampled at different time points, large genetic drift is expected, a sign that continuous reproduction has been taking place [12]. In genetic studies, these predictors make it possible to evaluate and distinguish between the different explanations for dry season survival.

### Approaches to studying malaria mosquito dry season survival and population rebounds

Two approaches, direct (ecological) and indirect (genetic) are used to study the seasonal dynamics of malaria vectors [13]. The direct approach mainly utilizes the mark-release-recapture (MRR) experiments [13], while the indirect approach relies on the genetic information from the samples collected. These include genetic diversity, population differentiation parameters, and temporal variation in allele frequencies, as a measure of genetic drift and  Ne [2, 12]. Results from indirect and direct approaches complement each other but are also usually different because the population size varies greatly through the year with estimates from the direct approach made when the population is near its maximum while that of the indirect approach is the Ne estimate which represents some sort of yearly average (harmonic mean) [63]. Several studies using direct or indirect approaches to investigate the different mosquito persistence mechanisms across sub-Saharan Africa have been carried out and are summarized in Table 1 with more detailed information for each study included in (see Additional file 1: Table S1).

### Computer simulations and dynamic models in population genetics to study mosquito persistence mechanisms

Malaria mosquito population genetic studies provide information about gene exchange between populations which is beneficial in making conclusions about the dispersal patterns of malaria vectors and in answering other ecological questions [70]. These patterns make it possible to predict vector competence, whose knowledge is critical in vector control, especially in understanding malaria vector genetic population structure and barriers to gene flow [70].

Computer simulations assist to assess the potential validity of the different hypotheses, determine which areas to consider for experimental studies, establish expected genetic signatures under different hypotheses and guide experimental work [71]. The use of dynamic models (used to simulate trajectories of change under different scenarios) is still in its infancy and is very important in highlighting several parameters such as changing temperature, mosquito dispersal, humidity, and mosquito size among others that contribute to vector dynamics observed in laboratory settings, semi-field conditions and the field [72]. The use of forward-time simulations (known to start from an initial population and follows its evolution from generation to generation) in population genetics to determine the origin of early wet season rebounds is promising and could be the most effective way to test between hypotheses [73]. Forward-time population genetic simulations play an important role in generating and testing evolutionary hypotheses that would be difficult to attain in laboratory settings because of the complexity of the process often known to be burdensome or even expensive [74].

The increase in population genomic data over the years has resulted in the use of more complex analyses using advanced simulation models [75]. These simulations are important for gaining an understanding of specific datasets used and in assessing and validating biological models [76], while evaluating the sampling properties of any statistics used on genome-wide association studies to compare the performance of different methods used [77]. Simulations usually allow for the inclusion of stochasticity in a natural way to investigate the entomological parameters relating to dry season ecology and movement behaviour which are still unclear in malaria vector species [71].

## Discussion

Over the years, several studies on the dry season persistence of *An. gambiae* species complex in sub-Saharan Africa have been carried out in the field, laboratory, and in-silico and have generated vast information and insights. How malaria vectors survive the long dry season remains unclear but could be associated with locality and niche-specific influences. Results from a study done on *An. coluzzii* populations in the Sahel and Riparian areas showed a difference in the aestivation phenotypes within and between the two environments, which signifies that there is a possibility that various populations of the same species have specific dry season survival strategies that depend on the strength and duration of the dry season in that locality [51]. That could be the reason why *An. coluzzii* populations of similar geographic origins undergo persistent local adaptations, which are also anticipated to be influenced by specific microhabitats [7, 26]. These adaptations may also be responsible for the fact that *An. gambiae*, a highly anthropophilic species has become both anthropophilic and/or endophilic [37].

Whereas some studies provide evidence for aestivation, local refugia, local or  LDM, repeating similar studies usually does not replicate the results [4], thus, the need to handle each geographical area independently because different populations may present different dry-season survival strategies depending on the strength and duration of the dry season. A study by Aboud et al. [78] in which *An. arabiensis* populations in South Sudan exhibited two phenotypic forms, one which was large and heavily melanized, while the other had the usual characteristics as found in other African settings (normal colour and size), results showed that the melanic form survived throughout the long dry season by partial aestivation [78], and was similar to populations found in *An. arabiensis* populations in Senegal [79]. The normal form, however, was inferred to persist by LDM [14], which was further confirmed by Atieli et al*.* [6]. Therefore, more studies that are geared towards comparing *An. gambiae* species complex populations from various environments especially where they occur in sympatry are important.

Using a combination of approaches, both direct and indirect in tandem because they complement each other could be a more credible way to not only understand dry season persistence mechanisms in the *An. gambiae* species complex, but also provide more insights into malaria vector population dynamics and how they affect vector control implementation. The marked mosquito recaptured at the start of the new rainy season (*An. coluzzii*) [4], and the *An. arabiensis* mosquitoes found at the end of the dry season [33] could either have survived by aestivation or as local refugia. Therefore, using both direct and indirect approaches in these studies could have resulted in more concrete and informed conclusions. Also, studies in genetic evolution and phenotypic plasticity combined with demography will assist in making predictions about population persistence in a changing environment. Population genetics using malaria mosquito genetic data will create a better understanding of the extent to which mosquitoes at the start of a rainy season are genetically distant from the previous season's populations [12].

Further studies could consider sequencing the whole *Anopheles* genome of mosquito populations from various areas in sub-Saharan Africa collected over several seasons to further elucidate the balance between longevity, reproduction and migration of the three species. Developing a modelling framework that could be extended into a spatial meta-population could also allow an assessment of the relative roles of different mosquito persistence mechanisms together with their environmental triggers. This will assist in predicting which genetic signatures are responsible for the different persistence mechanisms since the possible views that could explain each of them as mentioned earlier if tested using population genetic structure and temporal stability of genetic composition within populations have different expected outcomes [13]. Key parameters such as within-sample genetic diversity, between-sample genetic distance and temporal variance in allele frequency [12] could assist in making predictions based on each of the persistence mechanisms considered.

Using forward-time simulations in population genetics to determine the origin of early rainy season rebounds is promising and could be an effective way to test which persistence mechanism is more readily used by the three species. Forward-time population genetic simulations track complete ancestral information and are significant for deriving and testing evolutionary hypotheses that could be burdensome or expensive [74].

## Conclusions

Following studies to date, it still remains unclear which particular persistence mechanism(s) are responsible for the survival of each of the three species known to contribute the most to the malaria burden in sub-Saharan Africa. Using combined approaches (both ecological and genetic) is promising and has the added advantage of providing results that complement each other and provide more insights. This should reinforce the inexplicit theories that surround malaria vector population rebounds at the start of every rainy season. The clarity in this subject matter should also inform the effectiveness of the already existing and new malaria vector control tools which may include the use of genetically modified mosquitoes which constitute a new set of tools said to either replace malaria vector populations with introduced genes for refractoriness to limit malaria transmission or disrupt fertility genes and thus lower mosquito numbers to achieve vector population suppression.

### Supplementary Information

**Additional file 1: Table S1,** Studies that confirm or refute particular persistence mechanisms of malaria mosquitoes in Sub-Saharan Africa, hypothesis tested, results, and weaknesses.

## Data Availability

Not applicable.

## Abbreviations

*LDM*: Long-distance migration
*MRR*: Mark release recapture
*Ne*: Effective population size
*s.l.*: Sensu lato

## References

[1] Lemasson JJ, Fontenille D, Lochouarn L, Dia I, Simard F, Ba K (1997). Comparison of behavior and vector efficiency of *Anopheles gambiae* and *An. arabiensis* (Diptera: Culicidae) in Barkedji, a Sahelian area of Senegal. J Med Entomol.

[2] Taylor CE, Toure YT, Coluzzi M, Petrarca V (1993). Effective population size and persistence of *Anopheles arabiensis* during the dry season in west Africa. Med Vet Entomol.

[3] Touré YT, Petrarca V, Traoré SF, Coulibaly A, Maïga HM, Sankaré O (1994). Ecological genetic studies in the chromosomal form Mopti of Anopheles gambiae s.s. in Mali, West Africa. Genetica..

[4] Lehmann T, Dao A, Yaro AS, Adamou A, Kassogue Y, Diallo M (2010). Aestivation of the African malaria mosquito, *Anopheles gambiae* in the Sahel. Am J Trop Med Hyg.

[5] Service MW (1997). Mosquito ( Diptera : Culicidae ) dispersal — the long and short of it. J Med Entomol.

[6] Atieli HE, Zhou G, Zhong D, Wang X, Lee MC, Yaro AS (2023). Wind-assisted high-altitude dispersal of mosquitoes and other insects in East Africa. J Med Entomol.

[7] Dao A, Yaro AS, Dialloa M, Timbiné S, Huestis DL, Kassogué Y (2014). Signatures of aestivation and migration in Sahelian malaria mosquito populations. Nature.

[8] Charlwood JD, Vij R, Billingsley PF (2000). Dry season refugia of malaria-transmitting mosquitoes in a dry savannah zone of east Africa. Am J Trop Med Hyg.

[9] Connell JH, Slatyer RO (1977). Mechanisms of succession in natural communities and their role in community stability and organization. Am Nat.

[10] Faiman R, Yaro AS, Dao A, Sanogo ZL, Diallo M, Samake D (2022). Isotopic evidence that aestivation allows malaria mosquitoes to persist through the dry season in the Sahel. Nat Ecol Evol.

[11] Lehmann T, Hawley WA, Grebert H, Collins FH (1998). The effective population size of *Anopheles gambiae* in Kenya: implications for population structure. Mol Biol Evol.

[12] Lehmann T, Weetman D, Huestis LD, Yaro AS, Kassogue Y, Diallo M (2017). Tracing the origin of the early wet-season *Anopheles coluzzi* in the Sahel. Evol Appl.

[13] Simard F, Lehmann T, Lemasson JJ, Diatta M, Fontenille D (2000). Persistence of *Anopheles arabiensis* during the severe dry season conditions in Senegal: an indirect approach using microsatellite loci. Insect Mol Biol.

[14] Adamou A, Dao A, Timbine S, Kassogué Y, Yaro AS, Diallo M (2011). The contribution of aestivating mosquitoes to the persistence of *Anopheles gambiae* in the Sahel. Malar J.

[15] Sinka EM, Bangs JM, Manguin S, Rubio-Palis Y, Chareonviriyaphap T, Coetzee M (2012). A global map of dominant malaria vectors. Parasit Vectors.

[16] Wiebe A, Longbottom J, Gleave K, Shearer FM, Sinka ME, Massey NC (2017). Geographical distributions of African malaria vector sibling species and evidence for insecticide resistance. Malar J.

[17] Harbach RE (2004). The classification of genus *Anopheles* (Diptera: Culicidae): a working hypothesis of phylogenetic relationships. Bull Entomol Res.

[18] Coetzee M, Hunt RH, Wilkerson R, Torre AD, Coulibaly MBA, Besansky JN (2013). *Anopheles coluzzii* and *Anopheles amharicus*, new members of the *Anopheles gambiae* complex. Zootaxa.

[19] Coetzee M, Craig M, LeSueur D (2000). Distribution of African malaria mosquitoes belonging to the *Anopheles gambiae* complex. Parasitol Today.

[20] Afrane AY, Githeko KA, Yan G (2012). The ecology of *Anopheles* mosquitoes under climate change: case studies from the effects of environmental changes in East Africa Highlands. Ann N Y Acad Sci.

[21] Tandina F, Doumbo O, Yaro AS, Traoré SF, Parola P, Robert V (2018). Mosquitoes (Diptera: Culicidae) and mosquito-borne diseases in Mali. West Africa Parasit Vectors.

[22] Hay SI, Omumbo JA, Craig MH, Snow RW (2000). Earth observation, geographic information systems and *Plasmodium falciparum* malaria in sub-Saharan Africa. Adv Parasitol.

[23] Kamdem C, Tene Fossog B, Simard F, Etouna J, Ndo C, Kengne P (2012). Anthropogenic habitat disturbance and ecological divergence between incipient species of the malaria mosquito *Anopheles gambiae*. PLoS ONE.

[24] Lehmann T, Diabate A (2008). The molecular forms of *Anopheles gambiae*: a phenotypic perspective. Infect Genet Evol.

[25] Lefèvre T, Gouagna LC, Dabiré KR, Elguero E, Fontenille D, Renaud F (2009). Beyond nature and nurture: phenotypic plasticity in blood-feeding behavior of *Anopheles gambiae s.s.* when humans are not readily accessible. Am J Trop Med Hyg.

[26] Arcaz AC, Huestis DL, Dao A, Yaro AS, Diallo M, Andersen J (2016). Desiccation tolerance in *Anopheles coluzzii*: the effects of spiracle size and cuticular hydrocarbons. J Exp Biol.

[27] Magombedze G, Ferguson NM, Ghani AC (2018). A trade-off between dry season survival longevity and wet season high net reproduction can explain the persistence of *Anopheles* mosquitoes. Parasit Vectors.

[28] Lehmann T, Dao A, Yaro AS, Diallo M, Timbiné S, Huestis DL (2014). Seasonal variation in spatial distributions of *Anopheles gambiae* in a Sahelian village: evidence for aestivation. J Med Entomol.

[29] Huestis DL, Dao A, Diallo M, Sanogo ZL, Samake D, Yaro AS (2019). Windborne long-distance migration of malaria mosquitoes in the Sahe. Nature.

[30] Ramsdale DC, Fontaine ER. Ecological investigation of *Anopheles gambiae* and *Anopheles funestus*. I. Dry season studies in villages near Kaduna, Nigeria. 1970. WHO/MAL/70.735.

[31] Minakawa N, Githure JI, Beier JC, Yan G (2001). *Anopheline* mosquito survival strategies during the dry period in western Kenya. J Med Entomol.

[32] Huestis DL, Lehmann T (2014). Ecophysiology of *Anopheles gambiae s.l.* persistence in the Sahel. Infect Genet Evol.

[33] Omer SM, Cloudsley-Thompson JL (1970). Survival of female *Anopheles gambiae* Giles through a 9-month dry season in Sudan. Bull World Health Organ.

[34] Chisulumi PS, Nampelah B, Yohana R, Philbert A, Kweka EJ (2022). Diet and oviposition deprivation effects on survivorship, gonotrophic dissociation, and mortality of *Anopheles*
*gambiae*
*s*.*s*. J Parasitol Res.

[35] Depinay JMO, Mbogo CM, Killeen G, Knols B, Beier J, Carlson J (2004). A simulation model of African *Anopheles* ecology and population dynamics for the analysis of malaria transmission. Malar J.

[36] Koenraadt CJM, Paaijmans KP, Githeko AK, Knols BGJ, Takken W (2003). Egg hatching, larval movement and larval survival of the malaria vector *Anopheles gambiae* in desiccating habitats. Malar J.

[37] Holmes CJ, Benoit JB (2019). Biological adaptations associated with dehydration in mosquitoes. Insects.

[38] Canyon DV, Hii JLK, Müller R (1999). Adaptation of *Aedes aegypti* (Diptera: Culicidae) oviposition behavior in response to humidity and diet. J Insect Physiol.

[39] Benoit JB, Denlinger DL (2007). Suppression of water loss during adult diapause in the northern house mosquito. Culex pipiens J Exp Biol.

[40] Reidenbach KR, Cheng C, Liu F, Liu C, Besansky NJ, Syed Z (2014). Cuticular differences associated with aridity acclimation in African malaria vectors carrying alternative arrangements of inversion 2La. Parasit Vectors.

[41] Hidalgo K, Mouline K, Mamai W, Foucreau N, Dabiré KR, Bouchereau A (2014). Novel insights into the metabolic and biochemical underpinnings assisting dry-season survival in female malaria mosquitoes of the *Anopheles gambiae* complex. J Insect Physiol.

[42] Wang M-HH, Marinotti O, Vardo-Zalik A, Boparai R, Yan G (2011). Genome-wide transcriptional analysis of genes associated with acute desiccation stress in *Anopheles gambiae*. PLoS One.

[43] Fouet C, Gray E, Besansky NJ, Costantini C (2012). Adaptation to aridity in the malaria mosquito *Anopheles gambiae*: chromosomal inversion polymorphism and body size influence resistance to desiccation. PLoS ONE.

[44] Cheng C, Tan JC, Hahn MW, Besansky NJ (2018). Systems genetic analysis of inversion polymorphisms in the malaria mosquito *Anopheles gambiae*. Proc Natl Acad Sci USA.

[45] White BJ, Cheng C, Sangaré D, Lobo NF, Collins FH, Besansky NJ (2009). The population genomics of trans-specific inversion polymorphisms in *Anopheles gambiae*. Genetics.

[46] Gray EM, Rocca KA, Costantini C, Besansky NJ (2009). Inversion 2La is associated with enhanced desiccation resistance in *Anopheles gambiae*. Malar J.

[47] White BJ, Collins FH, Besansky NJ (2011). Evolution of *Anopheles gambiae* in relation to humans and malaria. Annu Rev Ecol Evol Syst.

[48] Touré YT, Petrarca V, Traoré SF, Coulibaly A, Maiga HM, Sankaré O (1999). The distribution and inversion polymorphism of chromosomally recognized taxa of the *Anopheles gambiae* complex in Mali. West Africa Parassitologia.

[49] Rinehart JP, Robich RM, Denlinger DL (2006). Enhanced cold and desiccation tolerance in diapausing adults of *Culex pipiens*, and a role for Hsp70 in response to cold shock but not as a component of the diapause program. J Med Entomol.

[50] Yang L, Denlinger DL, Piermarini PM (2017). The diapause program impacts renal excretion and molecular expression of aquaporins in the northern house mosquito. Culex pipiens J Insect Physiol.

[51] Yaro AS, Traoré AI, Huestis DL, Adamou A, Timbiné S, Kassogué Y (2012). Dry season reproductive depression of *Anopheles gambiae* in the Sahel. J Insect Physiol.

[52] Omer SM, Cloudsley-Thompson JL (1968). Dry season biology of *Anopheles gambiae* Giles in the Sudan. Nature.

[53] Huestis DL, Yaro AS, Traoré AI, Dieter KL, Nwagbara JI, Bowie AC (2012). Seasonal variation in metabolic rate, flight activity and body size of *Anopheles gambiae* in the Sahel. J Exp Biol.

[54] Huestis DL, Lehmann T (2014). Ecophysiology of *Anopheles gambiae s.l.*: persistence in the Sahel. Infect Genet Evol.

[55] Müller GC, Beier JC, Traore SF, Toure MB, Traore MM, Bah S (2010). Field experiments of *Anopheles gambiae* attraction to local fruits/seedpods and flowering plants in Mali to optimize strategies for malaria vector control in Africa using attractive toxic sugar bait methods. Malar J.

[56] Bell G, Collins S (2008). Adaptation, extinction and global change. Evol Appl.

[57] Denlinger DL, Armbruster PA (2014). Mosquito diapause. Annu Rev Entomol.

[58] Chevin LM, Lande R, Mace GM (2010). Adaptation, plasticity, and extinction in a changing environment: towards a predictive theory. PLoS Biol.

[59] Mamai W, Mouline K, Blais C, Larvor V, Dabiré KR, Ouedraogo GA (2014). Metabolomic and ecdysteroid variations in *Anopheles gambiae s.l.* mosquitoes exposed to the stressful conditions of the dry season in Burkina Faso, West Africa. Physiol Biochem Zool.

[60] Huestis LD, Artis LM, Armbruster AP, Lehmann T (2017). Photoperiodic responses of Sahelian malaria mosquitoes *Anopheles*
*coluzzii* and *An*. *arabiensis*. Parasit Vectors.

[61] Faiman R, Solon-Biet S, Sullivan M, Huestis DL, Lehmann T (2017). The contribution of dietary restriction to extended longevity in the malaria vector *Anopheles coluzzii*. Parasit Vectors.

[62] Wagoner KM, Lehmann T, Huestis DL, Ehrmann BM, Cech NB, Wasserberg G (2014). Identification of morphological and chemical markers of dry- and wet-season conditions in female *Anopheles gambiae* mosquitoes. Parasit Vectors.

[63] Touré YT, Dolo G, Petrarca V, Traoré SF, Bouaré M, Dao A (1998). Mark–release–recapture experiments with A*nopheles gambiae s.l.* in Banambani Village, Mali, to determine population size and structure. Med Vet Entomol.

[64] Constantini C, Li S, Torre AD, Sagnon N, Coluzzi M, Taylor TE (1996). Density, survival and dispersal of *Anopheles gambiae* complex mosquitoes in a West African Sudan savanna village. Med Vet Entomol.

[65] Hastenrath S, Polzin D (2011). Long-term variations of circulation in the tropical Atlantic sector and Sahel rainfall. Int J Climatol.

[66] Hastenrath S, Polzin D (2014). Variability of circulation and Sahel rainfall in the twentieth century. Int J Climatol.

[67] Salack S, Giannini A, Diakhaté M, Gaye AT, Muller B (2014). Oceanic influence on the sub-seasonal to interannual timing and frequency of extreme dry spells over the West African Sahel. Clim Dyn.

[68] Gatehouse AG (1994). Insect migration: variability and success in a capricious environment. Population Ecol.

[69] Drake VA, Farrow RA (1988). The influence of atmospheric structure and motions on insect migration. Ann Rev Entomol.

[70] Collins FH, Kamau L, Ranson HA, Vulule JM (2000). Molecular entomology and prospects for malaria control. Bull World Health Organ.

[71] North AR, Godfray HCJ (2018). Modelling the persistence of mosquito vectors of malaria in Burkina Faso. Malar J.

[72] Lunde TM, Korecha D, Loha E, Sorteberg A, Lindtjørn B (2013). A dynamic model of some malaria-transmitting *Anopheline* mosquitoes of the Afrotropical region. I. Model description and sensitivity analysis. Malar J.

[73] Hamad AA, Nugud AEHD, Arnot DE, Giha HA, Abdel-Muhsin AMA, Satti GMH (2002). A marked seasonality of malaria transmsission in two rural sites in eastern Sudan. Acta Trop.

[74] Ruths T, Nakhleh L (2013). Boosting forward-time population genetic simulators through genotype compression. BMC Bioinformatics.

[75] Adrion JR, Cole CB, Dukler N, Galloway JG, Gladstein AL, Gower G (2020). A community-maintained standard library of population genetic models. Elife.

[76] Escalona M, Rocha S, Posada D (2016). A comparison of tools for the simulation of genomic next-generation sequencing data. Nat Rev Genet.

[77] Carvajal-Rodriguez A (2008). Simulation of genomes: a review. Curr Genomics.

[78] Aboud M, Makhawi A, Verardi A, El Raba’a F, Elnaiem D-EE, Townson H (2014). A genotypically distinct, melanic variant of *Anopheles arabiensis* in Sudan is associated with arid environments. Malar J.

[79] Besansky NJ, Lehmann T, Fahey GT, Fontenille D, Braack LEO, Hawley WA (1997). Patterns of mitochondrial variation within and between African malaria vectors, *Anopheles gambiae* and *An. arabiensis*, suggest extensive gene flow. Genetics.

[80] Epopa PS, Millogo AA, Collins CM, North A, Tripet F, Benedict MQ (2017). The use of sequential mark-release-recapture experiments to estimate population size, survival and dispersal of male mosquitoes of the *Anopheles gambiae* complex in Bana, a west African humid savannah village. Parasit Vectors.

[81] Krajacich BJ, Sullivan M, Faiman R, Veru L, Graber L, Lehmann T (2020). Induction of long-lived potential aestivation states in laboratory *An*. *gambiae* mosquitoes. Parasit Vectors.

[82] Krajacich BJ, Huestis DL, Dao A, Yaro AS, Diallo M, Krishna A (2018). Investigation of the seasonal microbiome of *Anopheles coluzzii* mosquitoes in Mali. PLoS ONE.

[83] Mamai W, Simard F, Couret D, Ouedraogo GA, Renault D, Dabiré KR (2016). Monitoring dry season persistence of *Anopheles gambiae s.l.* populations in a contained semi-field system in southwestern Burkina Faso. West Africa. J Med Entomol.

[84] Gray EM, Bradley TJ (2005). Physiology of desiccation resistance in. Trop Med.

[85] Lee Y, Meneses CR, Fofana A, Lanzaro GC (2009). Desiccation resistance among subpopulations of *Anopheles gambiae s.s*. from Selinkenyi, Mali. J Med Entomol.

[86] Hidalgo K, Siaussat D, Braman V, Dabiré KR, Simard F, Mouline K (2016). Comparative physiological plasticity to desiccation in distinct populations of the malarial mosquito *Anopheles coluzzii*. Parasit Vectors.

[87] Davis J, Pavlova A, Thompson R, Sunnucks P (2013). Evolutionary refugia and ecological refuges: key concepts for conserving Australian arid zone freshwater biodiversity under climate change. Glob Chang Biol.

[88] Dao A, Yaro AS, Diallo M, Timbine S, Huestis DL, Kassogue Y (2014). Signatures of aestivation and migration in Sahelian malaria mosquito populations. Nature.

[89] Huestis DL, Yaro AS, Traoré AI, Adamou A, Kassogué Y, Diallo M (2011). Variation in metabolic rate of *Anopheles gambiae* and *A. arabiensis* in a Sahelian village. J Exp Biol.

[90] Lehmann T, Weetman D, Huestis DL, Yaro AS, Kassogue Y, Diallo M (2017). Tracing the origin of the early wet-season *Anopheles coluzzii* in the Sahel. Evol Appl.

