# Supplementary material for: Potential persistence mechanisms of the major Anopheles gambiae species complex malaria vectors in sub-Saharan Africa: a narrative review
Source: Malar J. 2023 Nov 7;22:336. doi: 10.1186/s12936-023-04775-0 (PMC10631165; doi:10.1186/s12936-023-04775-0)
Supplement: Supplementary file 1 — Additional file 1: Table S1, Studies that confirm or refute particular persistence mechanisms of malaria mosquitoes in Sub-Saharan Africa, hypothesis tested, results, and weaknesses. [file 12936_2023_4775_MOESM1_ESM.docx]

**Additional file 1**

**Supplementary Table 1: Studies that confirm or refute particular persistence mechanisms of malaria mosquitoes in Sub-Saharan Africa, hypothesis tested, results, and weaknesses.**

| **The method used & reference** | **Hypothesis tested** | **Results** | **Study Weaknesses** |
| --- | --- | --- | --- |
| Laboratory study [89] | Whether the long-lived state of *Anopheles gambiae* can be induced in the laboratory | In this study, the maximum lifespan of *Anopheles* mosquitoes was over 100 days representing maximum longevity compared to standard insectary conditions by 2.2-3.5 fold  Pushing the mosquitoes into exaggerated temperature/light conditions increased their chances of entering a long-lived state.  Using exaggerated photoperiods beyond what happens in Mali is likely to have pushed *Anopheles gambiae* to have similar longevity to that of *Anopheles coluzzii*, something studies carried out to date have never reported.  Mosquito wing sizes significantly increased In the dry season priming conditions  Reduced activity reduces sugar feeding and increases longevity | Laboratory colonies lose genetic diversity in a few generations  Laboratory conditions do not recapitulate all of the possible cues present in the field.  The lack of known markers of aestivation in *Anopheles* made it difficult to clearly confirm whether it really happened |
| Mark Release Recapture (MRR) [4] | Aestivation is responsible for the mosquito rebounds at the start of every wet season | Four days after the first rain, a marked female (*Anopheles coluzzii*) was recaptured. | Rare to recapture more than two or three mosquitoes a week after the release is done.  Some females break their aestivation more readily than others |
| Field survey studies in the Sudan [59] | Dry season survival mechanisms of *Anopheles coluzzii* | Vector density was too low during the dry season.  *Anopheles gambiae* females survived throughout the dry season. | Mosquitoes in this study were unable to lay eggs during the dry season. |
| Field survey studies [37] | *Anopheline* mosquito survival strategies | Two survival mechanisms were recorded; (1) Continuous reproduction happened in areas where aquatic habitats were available. (2) Mosquito embryos survived at least several days in soil with low moisture content.  One larva hatched from 124 dried soil samples, a sign that *Anopheles gambiae* embryos can survive at least several days in soil with low moisture.  At the onset of the rainy season, the dormant eggs are said to contribute to rapid population buildup, otherwise, the striking increase in population density cannot be explained solely by reproduction of remaining female adults in such a short time. | It is unclear whether anopheline mosquitoes prefer temporary habitats for oviposition or whether their larval density was very low due to predation and was difficult to detect in permanent habitats |
| MRR of *Anopheles gambiae* and *Anopheles arabiensis* in Mali [80] | Dry season survival of *Anopheles arabiensis* and *Anopheles gambiae* | Results showed no significant differences in either distribution or dispersal between the two sub-species.  A sizeable population of *Anopheles arabiensis* survived throughout the dry season. | MRR studies are impossible during the dry season when mosquitoes are hidden and hard to find.  There was no study to compare the results in relation to *Anopheles gambiae* |
| Mosquito Field collections in Kilombero valley [8] | Local refugia as a mechanism for dry season survival | Results showed evidence of mosquito dry season survival by refugia populations for all the three sub-species.  *Anopheles arabiensis* exhibited longer longevity compared to *Anopheles gambiae*.  During the dry season, *Anopheles arabiensis* exhibited lower longevity compared to *Anopheles gambiae.* | Distinguishing between absences which are as a result of poor sampling and those which are genuine is a challenge |
| Mosquito Field collections [14] | Study evaluated specific predictions of aestivation and migration hypotheses.  Separate predictions were formulated for each species and molecular forms increased the stringency of this multi-hypothesis test. | Only mosquitoes indoors were considered. This could be misleading given that sampling can aslo be done outdoors  Results concluded that *Anopheles coluzzii* (previously known as the M-form of *Anopheles gambiae*) survived the dry season by aestivation while *Anopheles gambiae* and *Anopheles arabiensis* survived by LDM. | The results were not consistent with the migration hypothesis because when mosquito density rose unexpectedly during the dry season, it could have represented the arrival of migrants from distant locations or locally aestivating mosquitoes hidden in the nearby shelters.  The source of the migrants is also puzzling since no known area in over a 100 km radius around the study area had a high mosquito density (the observation is therefore more related to aestivation than to migration) |
| Field collections [33] | Aestivation as the mechanism responsible for dry season survival. | Mosquito hotspots are a few local shelters because of dry-season-specific clusters or hotspots located closest to these shelters.  If mosquitoes are migrants from another village, they are unlikely to form hotspots but randonly diffuse into different houses.  Proximity to the mosquito sources is the key determinant for hotspot locations instead of unique house factors like structural design  Adjacent hotspots during the dry season exist at the village outskirts.  Dispersal of *Anopheles gambiae* species complex forms across houses doesn’t differ.  Results were comparable to other field studies to make the necessary conclusions | Hotspots were not predictable beyond being close to main larval sites, and were not the sources of the population rebounds  Only targeting mosquito hotspots misses the true sources of the population rebounds  Mosquito hotspots could shift if mosquitoes are deterred by the larval treatments. |
| Time series analysis (Field collections) [57] | Whether aestivation or migration is responsible for the mosquito rebounds at the start of every wet season | Mosquitoes were collected over a relatively long period of time (more reliable results)  The early wet season population by *Anopheles coluzzii* could have been because of their physiological differences.  *Anopheles gambiae* and *Anopheles arabiensis* experienced a lag in reproducing following a long dry spell.  During dry season, sporadic *Anopheles arabiensis* individuals were found, something consistent with local persistence as local refugia or aestivation.  This could also represent backcrossed hybrids between *Anopheles coluzzii* and *Anopheles arabiensis.* (Thus the need to use genetic data to check for different possibilities) | Not clear whether not collecting *Anopheles gambaie* and *Anopheles arabiensis* during the dry season was a weakness of the sample collection method or because of hidden shelters (local refugia/aestivation). |
| Aerial sampling of mosquitoes at 40-290m above ground level for evidence of windborne migration in four villages in the Sahel of Mali [34] | Evidence of LDM as a mechanism that supports mosquito rebounds at the start of every wet season | Results negated assumptions from previous studies that precluded that malaria mosquito dispersal doesn’t exceed 10km ^72,73^  The likelihood of capturing *Anopheles* species increased with altitude.  Millions of blood-fed malaria vectors frequenctly migrate over hundreds of kilometers  *Anopheline* mosquitoes that include among others *Anopheles coluzzii* and *Anopheles gambiae* were collected at heights between 40 and 290m above ground level.  Migration of malaria mosquitoes occurred over several nights.  Females out-numbered the males collected (4:1) | Species that were captured once could have migrated during one month. |
| Semi-field study (SFS) [72] | Evidence of aestivation and LDM as strategies for mosquito dry season survival | Setting up a laboratory colony of *Anopheles gambiae* from wildtype collections is difficult if the blood source is of an animal.  *Study results showed that Anopheles coluzzii* and *Anopheles arabiensis* aestivate while *Anopheles gambiae* could adopt a different dry season survival strategy such as LDM.  Host feeding preferences could be involved in causing species variation of the SFS given that *Anopheles coluzzii* and *Anopheles arabiensis* may be more succesful at exploiting calves for feeding compared to *Anopheles gambiae* | Aestivation and migration are the main mechanisms that explain variation in population dynamics.  Laboratory colonies lose genetic diversity within a few generations, thus not representative of the natural populations.  A low number of larvae was sampled for identification in order to avoid population depletion.  No species identification of adult mosquitoes was done which could have resulted in bias and narrowed the understanding of species dynamics.  The clay pots and building blocks as a means for providing shelter to mosquitoes are unnatural and give a biased representation of what happens in the natural environment.  The number of resting mosquitoes together with larvae found in breeding sites were lower than the number released, a sign that the conditions in the SFS may have not been optimal for survival. |
| The indirect approach: Using genetic data (current and long-term effective population size was estimated). [11] | Whether aestivation or migration is responsible for mosquito dry season survival | No annual bottlenecks occurred throughout the 7 and 9 years sampling intervals.  Large populations were maintained throughout the dry season.  Large populations could be maintained by individuals that are hidden with respect to sampling.  Large populations could be maintained by extensive movement of adults, thus low local level densities spread across a large geographical area | Reliable estimates of Ne are difficult to obtain for natural populations.  Overlooking significant differences in Ne may lead to erroneous estimates of gene flow and divergence time.  Violation of the assumptions considered could result in larger Ne values.  More information is required to assess the effect of constraints on Ne estimates.  Patterns of geneflow and their effect on Ne estimates need to be further evaluated |
| Indirect method: The effective population size of *Anopheles arabiensis* populations was calculated for various locations in West Africa [2] | To determine which mechanism is responsible for the dry season survival of mosquitoes | *Anopheles arabiensis* populations are maintained but with seasonal reductions.  The estimated Ne value was 2000 individuals, suggestive that *Anopheles arabiensis* in this region survive throughout the year, with seasonal reductions.  Survival could be in a physiologically altered state through specific adaptive mechanisms like gonotrophic dissociation, aestivation or reproductive quiscence. | It is unclear just how large an area should be to be included in the Ne estimates. The area to be considered depends on the distance from which the parents of an average individual would be drawn. There is need to use other molecular markers to confirm the results here |
| Indirect method: Use of genetic data. [13] | To determine which mechanism is responsible for the persistence of *Anopheles arabiensis* throughout the dry season. | Allele frequencies and indices of genetic diversity remained stable suggestive of the fact that large populations were maintained.  It would be nice to know how the results varry with the use of SNPs instead of microsatellite loci  To prove the scenario of extensive population mobility resulting in low local densities , moderate Ne values and very low local densities are recorded. | Genetic data cannot rule out that large populations survive in hidden shelters.  Numeric estimates of Ne appear higher across the dry season compared to the rainy season which reflects noise or could be because of a biological reason. It could also mean that mosquitoes aestivated during the dry season.  A few loci were considered which could have affected the preciseness of the results in determining the influence of inversions on Ne estimates.  Constraints on allele size or biased mutation rates on microsatellite loci found in non-coding regions whose biological function was considered neutral could have biased the estimates. |
| Using genetic data (SNPs) to establish the temporal variance in allele frequency signal from temporal samples collected during the rainy and dry season [12] | Aestivation is the main survival mechanism used by *Anopheles coluzzii* during the dry season | Two scenarios, those that depict aestivation or migration were considered.  Results support aestivation in *Anopheles coluzzii* during the dry season accompanied by LDM in the early late rainy season. | The results do not provide a solid fit to one or the other hypotheses necesitating reassessment of the underlying scenarios and consideration of additional ones.  Results from the two *Anopheles gambiae* samples show that genetic drift was not detected between them, which could be a consquence of lower power from smaller samples.  The median F statistic was used across loci to urgue that there was no drift for *Anopheles coluzzii*. The F statistic is naturally skewed and the median is always smaller than the mean. If the mean which is the standard Waple's method was used, then all combinations attained would have been finite Ne values.  Only two scenarios were considered and yet we have four succession mechanisms to compare.  For a follow-up study, there is need to use longer time-series and a greater coverage of the genome to narrow the uncertainty of the origin of the early rainy season *Anopheles coluzzii* population while evaluating the possibility of seasonal selection. |
